# “Compoundless Anaesthesia”, Controlled Administration, and Post-Operative Recovery Acceleration: Musings on Theoretical Nanomedicine Applications

**DOI:** 10.3390/jcm11010256

**Published:** 2022-01-04

**Authors:** Tyler Lance Jaynes

**Affiliations:** 1 Alden March Bioethics Institute, Albany Medical College, Albany, NY 12208, USA; jaynest@amc.edu; 2 Society for HealthCare Innovation (SHCI), Philadelphia, PA 19146, USA

**Keywords:** bioethics, blood–brain barrier, extracellular matrix, internal biomonitoring, nanotechnology, nervous system

## Abstract

Much research has been conducted on how patients may be served through new advances in perioperative anaesthetic care. However, adaptations of standardised care methodologies can only provide so many novel solutions for patients and caregivers alike. Similarly, unique methods such as nanoscopic liposomal package delivery for analgesics and affective numbing agents pose a similar issue—specifically that we are still left with the dilemma of patients for whom analgesics and numbing agents are ineffective or harmful. An examination of the potential gains that may result from the targeted development of nanorobotics for anaesthesia in perioperative care will be presented in this essay to help resolve this pending conflict for the research community. This examination should therefore serve as a “call to action” for such research and a “primer” for those for whom the method’s implementation would most directly impact.

## 1. Introduction

Anaesthesiologists and nurse anaesthetists or assistants have significant roles in a patient’s clinical care regardless of whether surgery is involved. Beyond possessing in-depth knowledge specific to the location of various arteries and veins, and more specialised knowledge for their area of expertise (namely, cardiothoracic, chronic pain, critical care, paediatric, regional, and research), they are necessarily required to do everything in their power to understand the patient’s status while under administration. Medical technologies have alleviated *some* of the traditional “guesswork” and real-time monitoring involved with these tasks. However, much is still left to chance because patients are fundamentally different on a biochemical level in each interaction, whether from one day under administration to the next or over a total lifetime of administration across health systems and institutions. For all that we have learned about the human body, and will continue to learn going forward, this fact alone is out of our control because our systems are continuously aiming to find homeostasis—entailing that individual nutrition excess and deficiencies are in constant flux to keep internal systems regulated and sufficiently functional.

In this vein of thought, nanoscale-based medicine is seen as the “next frontier” whereby clinical advances are expected to improve patient well-being and care substantially. Already, we are seeing the direct impact of nanoscopic technologies via the means whereby the SARS-CoV-2 virus is being combatted [1,2,3]. Similarly, nanoscale liposomal package delivery systems for analgesic delivery show marked improvements to patients’ perceived quality of care [4,5,6] as one of many techniques aiding pain management [4,7,8]. Nevertheless, with the difficulties involved with the manufacturing of nanoscale robotics, the benefits currently seen from nano-scaled science and engineering can only come from advances in manufacturing that are easier to produce on a massive (industrial) scale. Hence why much research on industry-scale advances in nano-scaled science are oft avoided until consistency in production is achieved, along with greater understandings of the toxicity that results from nanoscale technology implementation and other such health and quality concerns [9,10,11,12], and why the field retains a relatively small market share in non-research medical expenditures internationally (SARS-CoV-2 vaccines notwithstanding).

Aspects of nanoscale potentials are far from unknown in the public sphere, even in the face of these challenges. Internal health monitoring with nanoscale devices, for example, is far from a “new” concept to many. Opinions have become mixed among those aware of nanotechnology’s limitations as to the permanent presence of “nanobots” within the human form because of science-fiction and educational scientific media (the latter of which may sometimes be more hype than pure fact). Of course, this is just *one* prominent branch of development for how real-time internal diagnostics might be carried out on a continuous, remote basis. Moreover, while research has displayed our ability to manipulate cell membranes to create biosensors [13,14,15,16] and maintain membrane integrity when attempting to deliver pharmaceutical compounds [17,18,19], a key challenge for (nearly) non-invasive medicine is the ability of nanowires, nanosensors, or other such nano-scaled probes to manipulate the extracellular matrix (ECM) or blood–brain barrier (BBB) in a way that does not leave permanent structural damage.

There are methods whereby our access to our internal structures can be made wholly non-invasive—such as through the oral or nasal cavities—so it cannot be stated that no such avenue exists presently. Insofar as efforts continue to be made in the direction of health “wearables” or “insideables” [20,21,22,23,24], there will be a public desire to ensure that considerations are being made for the minimisation of damage through the use and maintenance of devices and implements that must enter the body through the ECM and other tissues or are otherwise implanted for extended periods. Even for “necessary” intravenous fluid or drug administration in clinical settings, let us not forget that patients may believe *any* implement entering their body is “invasive” even if it is not clinically understood to be so to practitioners, legal experts, and ethicists. Nor that they may wish to minimise the contact they have with medical personnel for a range of reasons outside of their current medical insurance coverage, which may result in a need to ensure that devices can be self-maintained by their nature or easily maintained by the patient.

While these may seem “obvious” concerns to advocate for and be wary of, the need for bioethical discourse on the subject *alone* should be evidence enough that even the “obvious” facts of medical practice can be forgotten (even accidentally) while a given technology is under development [23,24]. This reality is especially significant for a device that can potentially interface directly with the human central and peripheral nervous systems (CNS and PNS, respectively). The potential to directly interface with the CNS or PNS to detect the origin of a “pain” signal, or similarly to restrict signal transmission to create a “compoundless analgesic,” may be tempting enough innovations to leave common ethical norms by the wayside for their realisation.

Coming back to the origins of this article, however, the targeted development of a biomonitoring and substance administration system through the implementation of nanotubes, nanowires, nanoprobes, and other nanoscale architectures may yet reveal a new avenue whereby anaesthesiologists and clinical physicians can further hone their attention to perioperative care without requiring the use of the analgesics of today’s clinical settings. While some in the field may assume that the future of anaesthesiology or clinical medicine lies in the development of new protocols whereby something akin to personalised medicine is attainable to serve patient populations better, or through the development of hypodermic needles and other surgical instruments less prone to microbial contamination, these are but ideas that are bound to the practices that are in everyday use. Because they are bound to practices that have proven effective in routine patient care to date, there is a limitation to the relative level of “good” that can be further attained because of this narrow line of research. As such, a theoretical exploration of new technological avenues for medical device advancement is required to stimulate those who wish to advance medical sciences to better patient experiences so that growth in standardised perioperative procedures can (potentially) occur.

### 1.1. Background

This work relies heavily upon ideas originally proposed by the author in conference venues in the USA, Europe, and Asia—which drew inspiration from Japanese media depicting the phenomenon of full-dive virtual reality gameplay utilising high-frequency microwaves [25,26,27,28,29,30,31]. Given the difficulties for microwaves or analogous radio frequency (RF) waveforms to perform the tasks depicted in Reki Kawahara’s storyline on account of the effects of prolonged exposure to the human from RF radiation (and other issues related to the use of radiation to measure neural activity) [32,33,34,35,36], this author proposed to develop facsimilia to the four devices presented by Kawahara—though with the use of nanorobotics tethered to wire-embedded devices before leaping remotely dictated independent locomotion within the human form.

The rationale for pursuing the ultimate development of such facsimilia was spurred by the medical applications described by Kawahara for the devices presented in his story [27,30,31,37], with an emphasis on his depiction of one device as a machine capable of inducing an analgesic-like response which also served as a comprehensive tool for long-term patient care [27,30]. Although that specific device is one of many posited to exist in futuristic societies in science-fiction media, the ability of it and its “parent” device to block signal transmission between the CNS and PNS (alongside its ability to allow physicians and loved ones to interact with otherwise “locked-in” patients through the virtual embodiment of a digital avatar) [25,27,29,30] were enough to prompt an in-depth exploration to how these same effects can be attained through nanoscale device research. Where other media have similarly picked up on elements of Kawahara’s work or ideas preceding it in the litRGP or *isekai ten’i* (異世界転移、いせかいてんい) genres focused on augmented or virtual reality internationally, this author’s assumptions were (and remain) that users will demand such facsimilia of augmented/extended/virtual reality (AR, XR, and VR, respectively) devices. Such artefacts may likely use those developed by Oculus, HTC Vive, and a myriad of other traditional and emerging electronics and virtual-reality-specific companies as progenitor models, and will be demanded because of how this technology’s capabilities are depicted in these literary and televised mediums.

Hence, the actual means whereby these theoretical concepts become a reality may be within developers’ grasp. Outside of research ethics and developmental expenditures, the only barrier is time. XR technologies may spontaneously gain “full-dive” capabilities that do not require physical motions if collaborations are established between work being conducted on neuroprostheses or other technologies that would feasibly translate our brain’s commands into actions performed by virtual avatars. Hypothesising that the same methods used to establish a “compoundless analgesic” reaction in the body via nanoscale technological manipulations of biological processes can bridge the gap between physically and mentally driven VR interactions, it can be rationally asserted that caution and education towards this branch of technological development be enacted to poise practitioners and researchers for the ethical and social issues that will arise from this effort before it becomes a reality. Such is the traditional remit of bioethics research [38] and a necessary step as new generations of engineers reared on this media gain professional accreditation and authority.

## 2. Materials and Methodology

As depicted in Section 1.1, the concept grounding the theoretical system depicted in the Introduction arose as a practical means to combine PNS/CNS interactions and targeted biomonitoring and compound delivery. At present, there is no constraint surrounding the “final” form such a device would take because there are benefits and detriments that arise from implementing a system of this nature in artefacts of all shapes and sizes. Similarly, there are no formal specifications on the optimal mixture of nanostructures that may be implemented in this system because they will have to be optimised to fit best the “hub” device controlling their function. Instead, the purpose of this section is to provide some visualisation on the elements that may feasibly comprise artefacts developed for the express purpose of multimodal internal biomonitoring and signal control utilising nanoscale structures so that their potential benefits and detriments can be explicated later in this essay.

For instance, this system may take the form of the quotidian compound anaesthetic machines that many of us internationally use daily. Given the multifunctional nature of compound systems, the resulting care that may feasibly result from highly targeted administration of nerve blocks or compounds (e.g., minerals, liquids, proteins, fats, carbohydrates) and real-time biomonitoring of cellular activities, blood pressure, and like processes, would remain as a significant improvement to the overall anaesthetic care we can deliver today. Consequently, such a device would require anaesthesiologists and other anaesthesia-administrating personnel to administer a greater range of care than currently required because of this increase in technological sophistication. Whether or not this extended range of care will require more from anaesthesia-administrating personnel depends on the related gains that arise from other areas of medical technology, including innovations in AI-drive automation, computer vision and robotic caregiving capability, AR/XR/VR interoperability via Internet connectivity, and quantum physics—but this will be expanded upon later.

Separate devices may feasibly undergo development, where the neurological interfacing and compound delivery systems are divided into unique, standalone artefacts. These, in turn, may feasibly become hyper-focused to treat specific tissue structures or sections of the human form if necessity demands such specialisation. However, such a pursuit would inevitably serve as a blow to the equitability of care and thus the implementation of this technology in environments lacking funds for conversion—namely, in rural clinics, developing nations, and areas of conflict. Conversely, the cost savings from implementing these devices would eventually make independent setups desirable for smaller clinics if we assumed that these standalone artefacts can be manufactured to take up less space than the compound machines in use today.

Depending on the part of the human body such a system needed to be deployed upon, the system could likely take up a wide range of forms—such as a sphygmomanometer cuff, pulse oximetry probe, or even an examination table or long-term care bed. The sole requirements for the system would be to have a housing site for the nano-scaled architectures and a method whereby the system can replace damaged or lost nanoscale components (beyond a method to ensure nanoprobe and nanowire sterilisation between uses, should that prove to be necessary). Implementational considerations include the potential need to coat the nanoscale components in unique proteins, enzymes, and other receptors which match a given patient to further aid the immune system’s acceptance of architectural components as part of the body, although they are not limited only to this topic. That being stated, the requirement of such unique coatings would ultimately result in the system being considered ultimately non-viable due to the preparation time necessary between patient use if it is not equipped with the ability to self-produce said markers and discard them between uses. This consideration is similar to that which is generally found in personalised medicine, though it is likely more difficult to ignore due to blood-borne ailments and, as such, requires directed experimentation and observation to understand more comprehensively.

Whether standalone devices can feasibly be made portable, however, is ultimately a question of how quickly the computational system attached to it can be miniaturised—beyond those considerations that are attached to the “need” to coat nanostructures in proteins that will not appear “foreign” to the patient’s body. Larger systems, as currently employed in state-of-the-art facilities, may be capable of employing a “hub” device that manages several hundred nanoscale probes if enough of the tasks performed by these sensors are simple or require little processing power to translate into machine-readable information. However, real-world research and programming must be undertaken if these “hubs” house thousands or tens of thousands of tethered probes that can perform more sophisticated monitoring or other related tasks. After all, the computational capacity required by these machines to enable “autonomous” nano-scaled structure movement is realistically proportional to the number of structures connected to the “hub” they would be anchored to—though more research in this area will need to be conducted to verify whether a logarithmic, exponential, or linear relationship is the norm across programmes enrolled for this task and whether specific programming languages influence these relationships (existing or newly fashioned for this task) in any statistically-meaningful manner.

The short-term operational capacity of any of these artefacts will depend upon whether the devices rely upon traditional computational means or upon those that would be based on quantum or optical mechanics. While we may accept that conventional computational methods are the easiest to work with because we already possess the infrastructure to develop artefacts with this methodology, it is difficult to refute the benefits that arise from the implementation of non-conventional methods that are still in development—or which exist in theory alone [39,40,41,42]. At the same time, there is the reality that interoperable interfaces between conventional and non-conventional systems present another challenge that needs to be addressed—namely, in how software interacts across different logic systems, input/output modalities, and new file types which quantify emerging informational storage derived from non-conventional processes. This series of unknowns brings the need to determine whether current computer language systems can handle these new tasks, or whether a new singular or set of languages that existing ones inevitably phase into must be developed as implied in the prior paragraph.

Other considerations for artefact design here should include the means employed by nanoscale probes and sensors to collect and transmit data, as these will likewise be influenced by the type of computation and software language utilised by the “hub” systems they are attached to or from which they are deployed. Optical computation, for example, may require that nanostructures are shielded to prevent photonic damage to patients and physicians alike. Nanostructures that send data in wireless manners will need to ensure that their signals can only be collected by their “hub” structure to limit the potential for nefarious actor intervention and information collection by other wireless devices not intended as authorised recipients of these data. Whether this process can be industrially standardised and prevented from being replicated in other wireless communications is a conversation that will need to occur, as it is likely to cause a significant conflict between companies internationally that cannot be handled by legal systems or international treaties that require nation-state ratification. Consideration is less vital for the means whereby targeted compound delivery is employed, primarily because there are fewer factors at play for that specific process, though there will need to be consideration given to the means whereby pressure is maintained in nanotubules to prevent drastic changes to blood pressure within patients and structural damage to nanoscale structures more generally.

Whether this device could jointly be used to regulate blood pressure and prevent patients from fibrillating during surgery is another area of research that would need to be undertaken with careful consideration. The ability to enforce a specific heart rate, blood pressure, or fluid flow via nanotechnology would likely be seen as research areas too dangerous to pursue or make public under the lens of new and emerging bioterrorism modalities without counteraction strategies being developed beforehand. Similar concerns arise with its use as a coagulation agent and in conjunction with any function envisioned herein. However, they are far enough into the future that countermeasure research can be stipulated as a requirement for any progress towards developing any system so far discussed. The more significant concern then becomes whether Defence Ministries or Departments attached to autonomous or international states must take up the mantle of this research, or whether civilian contractors are entrusted with this task under strict organisational oversight regimes. That being stated, the public supposition that these modalities are feasible is already a signal that such counteraction measures need to be pursued immediately by international security agencies (if such work is not already underway).

Moreover, of course, we cannot ignore the material composition of these nanoscale structures. Where composition will vary depending on the computational medium used for the device, and whether they employ biomemristive or other biomimetic components, there is no simple answer as to the dimension the structures should be built-in (e.g., singular or multi-dimensional) and the elements that should be sourced for their development. Such discussions are better suited for the developmental environments of these modalities than a theoretical discussion such as that presented here, however.

## 3. Discussion

With the discussion of artefacts that may feasibly interact with the PNS/CNS in ways that would reduce or remove the need to manufacture opioids or other anaesthetic compounds, it is important to iterate and focus on the ethical concerns surrounding its development and implementation. Beyond the reality that commercial utilisation of nanoscale devices is limited—if not altogether non-existent in many markets—at present, there is the aforementioned concern that interest in this sphere may need to be left in the hands of code-word clearance projects alone until industrial standardisation or legislation can be formulated to protect against its use as a torture device or weapon of mass harm.

Given the trends arising from the EU’s recent release of legislation on AI, or more specifically, how other international legislative bodies have now begun to draft legislation that can match it, the burden of such efforts may have to fall upon that body’s shoulders. While many in the field may look to the Food and Drug Administration (FDA) in the USA as a guidepost for projects of this nature, given their focus on consumer protections for medical and non-medical devices alike, it is challenging to state whether the FDA can maintain political neutrality in that nation’s current climate. This statement is made with the understanding that funding—military or otherwise—for research on nanoscale devices that can pierce the ECM is likely something that would be difficult to secure given the lack of bipartisan cooperation to settle on yearly operational budgets and the like, and that many other nations excel in the field of nanotechnological research with or without independent laboratory aid from American scientists.

More importantly, there remains the reality that international cooperation will be needed to ensure that nanoscale, multimodal internal manipulation, monitoring, and administration can be effectively implemented and monitored. For as much good as the World Medical Association or World Health Organization does for our community, specifically with the drafting of documents such as the Declaration of Taipei and tracking infectious diseases, they are insufficient to establish treaties of a scale comparable to the Geneva Conventions. Between the advances taking place in artificial intelligence systems (AIS) more generally [23,42,43,44,45] and the proposed advances to nanoscale medicine described here, simply generating a “community spirit” that is dedicated to self-regulation—as is the case for the “DIY Bio” crowd [46,47,48]—is insufficient to ensure public safety. Having “faith” in the “good intentions” of others can only go so far when the potential for technological misuse reaches a certain threshold, and consequences need to be put into place as a deterrent for that end.

On a lighter note, some steps can be taken by the biomedical community that can serve as an intermediary “stop-gap” between international regulation and the state of the field as it exists today. Before a discourse on that subject can be broached, however, attaining an understanding of the progress that remains between our current research and that which still needs to be conducted is prudent to help frame the recommendations that will be brought forth from that narrative.

### 3.1. Feasibility of Envisioned Systems

For clarity, there have been several advances in recent years that would allow for the development and testing of artefacts utilising multimodal internal biomonitoring and signal control, including administering an anaesthetic-like reaction that does not rely upon pharmaceutical compounds (also understood as “compoundless anaesthesia”). These include a rapid acceleration into dermal structure research, construction, and tactile sensor development for artificial skin [49,50,51,52,53,54], and the general computational capabilities of AIS and sophistication of applications to translate highly complex data into machine-readable information [23,42,43,44,45,55,56,57]. Such research has revealed a more comprehensive understanding of cellular membrane viscosity [58], and may yet lead to a better understanding of how membrane viscosity impacts cellular communication and transportation alongside ECM integrity and methods whereby ECM connections can be “harmlessly” parted to allow for nanoprobe entry. Of course, these advances must be bracketed with the understanding that AIS are still fallible in many of the areas of use they are implemented in [55]—along with the disclaimer that medicine remains difficult to generalise on account of each human’s innate biochemical differences.

Whether our expansion of knowledge into ECM manipulation can be translated into “harmless” BBB piercing is a separate question, however—especially where concerns remain as to the “medical necessity” to directly access cortical or subcortical tissue if neural access can be attained by PNS interfacing in the spinal column. It should also not be forgotten that there may be differences between the integrity of the ECM and the underlying fat structures that support skin tissue, and that such differences may further delay progress towards a “harmless” entry of nano-scaled architectures into the human form. These differences necessarily include an implicit understanding that a certain percentage of fat tissue is necessary for life, which may require that public educational efforts be revitalised to counteract the backfired attempts of USA-based companies to promote “healthy” lifestyles that have created skewed perceptions of body image and “normal” body fat percentages.

Furthermore, questions remain as to whether external haematic nutritional manipulation can be just as specific in targeting areas of deprivation within brain tissue as it may be in other areas of the human form. These questions are, in part, spawned due to the uncertainty surrounding the safety of manipulating the BBB, and whether its structure can be harmlessly manipulated with the same methods that would be employed for ECM penetration. They also arise because of the differences in function between the BBB and ECM, and traditional concerns regarding the precision delivery of compounds across the BBB and underlying support structures even if they are proving effective in recent research [59,60,61]. While the resolution of these questions and concerns may *seem* simple to address, reality reminds us that the procedures set out in internal review boards and medical ethics committees necessitate that proper safety measures are implemented for research of this nature because of the potential for harm that may result in permanent BBB damage or miscalculations into hyper-tuned haematic manipulation.

Given the concerns that arise from developing a system that can feasibly connect to nerve fibres and receive information from (or direct commands to) them if it existed as an implanted device, it might yet remain inadvisable to advocate for the creation of any device as described. For clarity, it is believed that having a direct connection to any nano-scaled probe is the safest route to pursue on account of security issues that follow with the use of wireless communications between the probe and command system. After all, the most significant issue with wireless communication is that far too little can be done to restrict a system’s access to the communications generated between the implanted system and the command or “host” system using current modalities. Hence, it is not believed that a device that can be administered orally or through other such entry points is ethically justifiable at this point in time for the collection of data utilising relatively non-invasive means—precisely because there is no practical pathway whereby nanobots or nanoprobes connected to command systems wirelessly can assuredly be deactivated should they become infected with a computer-based virus or are otherwise “hacked” by malicious actors.

There are still concerns regarding whether neural signal isolation is *enough* to ensure that the presentation of an anaesthetic-like reaction is expressed in a given subject. Expressly, it is generally understood that analgesic compounds are effective because they can trigger “numbing” reactions to several organ structures simultaneously while maintaining regular blood flow through the affected areas. Whether reactions to “pain” are limited to nervous cell transmission alone is a subject that requires objective observation and verification, however—which shall be discussed in more detail in Section 3.2.

### 3.2. Anaesthesiology in the Context of Neuroethics

A topic of intersection here concerns the ethical responsibilities held by anaesthesiologists of all stripes, which may get overlooked in circumstances or situations unrelated to the “surfacing” of patients during an intensive surgical procedure. Similar to researchers who specialise in the study of pain management, the composition of the depicted artefacts herein will require that anaesthesiologists confront the “nature” of pain on a more detailed level and become involved in the phenomenological, existential, and metaphysical studies that have grown in the realm of neuroethics research if they are not already engaged in those discourses. That is not to say that the author expects philosophers to become pseud-specialists in the art of anaesthesiologic administration or that administrators become pseudo-specialists in the other direction. Instead, the implication here is that collaborations between these two fields of study will become an integral part of developing, researching, and implementing the depicted artefacts.

As mentioned, many ethical issues require consideration when contemplating a device of this specific nature beyond those alluded to in Section 2. Before considerations can be made as to the viability of ECM or BBB penetration without altering the structure’s overall integrity, we must first consider how such a device might proceed through clinical trial oversight. Simulations or live experiments, which simulate whether fluids are expelled upon initial nanostructure penetration of the skin, or perform similar tasks, may be conductible. In the case of live experiments, this may be conducted using necrotic (implying dead, not decaying) tissue from human or animal cadavers or that which is otherwise “printed” and not connected to a living organism. While these may seem trivial experiments to conduct, as they do not indicate whether the flesh would be undergoing a sensation of pain or discomfort, they are necessary nevertheless because they display the ability for nanostructures to be employed in a manner that does not damage the integrity of the ECM. Similar experiments may feasibly be conducted for all manners of tissue structures and organs found in the human body as necessary, but again point to the need to scientifically verify that their integrity is not compromised in a clinically significant manner.

Nevertheless, these experiments will face many challenges when attempts are made to prove this same level of non-compromission with tissue that is “live.” Notwithstanding the arguments that will be made in respect to the avoidance of live-animal studies until evidence can be produced as to the lack of “harm” that would befall them, there are emerging ethical concerns that surround the use of “live” organoids [62,63,64,65,66]. Contrary to the assumptions of many researchers, there are not many studies that focus on the ethical implications of “pain” phenomena in nerve fibres or cells implemented in organoid cultures outside of those focused on cerebral organoids. This lack of emphasis is most likely because the phenomenological inquiry into the ability of these organoids to develop a “conscious” state of being is considered more significant and pressing, and therefore deserving or requiring a different set of considerations for institutional research specific to the creation of neurological tissues.

That being stated, there will nevertheless be institutional review boards that question whether the artificially created nerve cells and fibres can experience pain—and whether the cellular experience *alone* is enough to reject the pursual of a study such as this. The barrier to a study that would test the perception of pain via nano-scaled architecture penetration of the ECM may become the presence of pain in nerve cells and fibres when disconnected from processing centres, which can feasibly interpret the nature of those signals. This concern is presented assuming that localised cellular reactions may be theoretically interpreted as a “processed” response to a given “pain” signal or may otherwise generate reactions that could trigger a sympathetic neurological reaction.

Parallel to this concern is whether the use of analgesics to mitigate “pain” responses from nerve cells and fibres alters the data necessary to prove that ECM penetration by nanostructures does not cause undue stress to the patient and targeted tissues, tissue structures, and organs. While we might understand the electrochemical response that generally follows “pain” sensations, we do not currently understand whether there are differentiations between pain caused by a change in temperature, tactile sensation, or structural damage to tissues. Furthermore, there currently exist no data on the “static” state of nerve cells and fibres more generally—mainly because there is no such thing as a “static” state within our individual forms—so any such evidence that does exist necessarily needs to be re-examined with this reality in mind if these assumptions were not built into the research hypotheses. Whether because of minute muscle movements to maintain balance, the cellular-level transport of materials and movement of microscopic organisms within our form, or the regular motions that accompany the functioning of the bloodstream, our bodies could be said to be in a constant state of motion. This set of minute processes, in turn, requires that our interpretation of nerve-based signals be skewed towards this reality. The problem, however, is that it will take a significant amount of research across age, gender, and ethnic groups to attain a standard baseline for what each individual signal “means” to our central processing systems—if, of course, they can be delineated into unique categorisation schemes. It is very likely that these responses will be just as unique as individual biochemistry, and that few generalisations can be extrapolated as a result, so we cannot be discouraged if our attempts to standardise these indicators not realised.

If a case can be made that a disconnected nerve cell or fibre cannot effectively “feel” pain because it has no system whereby the signals it sends can be received, research may then be able to proceed using manufactured dermal systems. However, considerations will still need to be made as to how nanoprobe interactions with nerve cells are to be engaged. For example, it might be simple enough to interact with unmyelinated sections of a nerve axon but not so simple to interface with the myelinated sections because myelin sheathe integrity is significant for the accelerated transfer of information within the nervous system. If interactions with the myelinated sections of a nerve axon prove to degrade its overall integrity over time, and attempts to re-myelinate damaged sections prove futile, the system’s effectiveness will proportionally be compromised. Where short-term studies will be insufficient for this task, it would likely take years for this stage of research to provide satisfactory results—especially if requirements are made to test the nerve cell at various stages of its lifecycle, or computer simulated studies cannot determine the relative functioning of nerve cells and fibres in advanced ages because of the endless number of environmental factors at play.

Moreover, none of this begins to touch on the need to examine how blood pressure, heart rate, and neurotransmitter presence impact the recordings received by the nerve cell or fibre. Assuming that the system would not be implemented solely under “rest” conditions, research will need to be conducted to test how nervous system interactions and control are impacted under trauma-like conditions or those present after a patient undergoes various levels of exercise. Because this system is not mainly being developed to induce a static-body state to enable an individual to engage in a “fully” immersive virtual-reality experience [25,26,27,28,29,30,31,67], data on the various stages of excitation and homeostasis that a human body can be subjected to is necessary for us to understand how to better ensure the safe usage of the system under these conditions. This data consideration, of course, includes how neurotransmitters may interfere with our attempts to prevent signals specific to “pain” or “heat” from being transmitted, which may realistically interfere with our ability to utilise the system as a surrogate for compound-based anaesthesia.

Considering the result of the posited systems, the design and research goals are ultimately to find a method whereby probes can effectively detect the source of a “pain” signal by connecting to nerve fibres in the CNS and PNS while also providing an avenue for physicians to block the transmission of “pain” signals in highly localised areas for surgical purposes or HIV treatment. Whether this research leads to the potential for internal muscle stimulation to occur through its use, the immediate goal of developmental research will be focused on the differentiation of PNS/CNS signals and their influence on intracellular communication and material transfer. Barring the development of beds that absorb materials excreted through the skin (including dead skin cells, hair, and sweat) and apply targeted electrical stimulation to prevent the development of bedsores or other muscle degradation [25,67], the use of targeted dermal and muscle stimulation through ECM-breaching nanostructures may further prove to serve those requiring long-term medical attention. While this would potentially reduce the number of interactions a patient may have with their care team, it has the hidden benefit of reducing the number of staff needed to physically manipulate patients’ bodies—which does take some measure of strength and energy, particularly if they are in a non-responsive state. This latter point is significant if our medical systems see a continued decline in overall personnel after the SARS-CoV-2 pandemic, particularly because there will be fewer “young” people in developed nations to perform the tasks necessary for elder care per today’s population modelling predictions and trend analyses.

These are just some of the baseline connections that will need to be made between the bioengineering researchers that partake in applying the ideas theorised herein and will significantly transform how research in anaesthesiology and neuroethics will progress. Given the need to ensure sufficient expertise from those medical practitioners, legal scholars, engineers, and ethicists that undertake this work, an educational or certification regime must therefore be established that promotes the synergy between these varied disciplines. This regime may include implementing specialised AIS programme developmental courses, AR/XR/VR sandbox environments that enable magnified architectural design without wasting material resources, or other similar elements that would facilitate ethical and sustainable research. How that is ultimately conducted is beyond the scope of this essay but is critical to note because the early adoption of such regimes stimulates the defence, governmental, and industrial backbones that will ultimately bring approved devices into the marketplace.

### 3.3. A Brief Discussion on Invasiveness and Long-Term Patient Health with Implantation

The literature surrounding the nature of “invasive” procedures is extended and connected to case law that has helped to shape the nature of medical disclosures related to the delineation that exists between “invasive,” “minimally invasive,” and “non-invasive” techniques. Furthermore, because the integrity of the ECM/BBB without a nervous system or immune response after nanoprobe penetration is achieved blurs the line between these techniques, it is important to reiterate some of these issues and those that relate to the presence of a foreign, mechanical object within the patient’s body because they are connected to the state of nanomaterial toxicity testing today.

Beyond the recovery time that is associated with a procedure regardless of its “invasive” nature, there are concerns that prolonged implantation may lead to increased risk of microbial or infection—outside of the reality that the internal environment of the human body is corrosive to devices heavily reliant upon metal for operation and electrical signal conduction. Even if metals such as silver see increased usage in medical implements [68,69,70,71], the reality remains that there are harms that follow overuse of this and related metals—beyond having skin pigmentation change to a bluish hue [72,73,74]—as is the case with any substance that disrupts the homeostasis of a cell or complex organism. Where there are long-term dangers that arise with implanted devices that specifically involve their maintenance and deactivation, the development of a minimally invasive device helps ensure that repetitive surgeries are unnecessary (or, at least, less necessary overall). Hence why there has been so much emphasis herein on moving away from the implantation of nano-scaled devices that can communicate wirelessly beyond the aforementioned security concerns those devices pose, and why long-term nanodevice integration into the bloodstream of a patient may be too expensive over their lifetime to implement.

Furthermore, emphasis has been placed on avoiding damage to the ECM and BBB under the assumption that it would serve multiple purposes beyond preventing the creation of a wound that may potentially become infected by harmful air- or skin-borne bacteria, viruses, or other microscopic organisms. First, it may feasibly prevent standard defensive mechanisms from being engaged within the human immune system—thereby effectively preventing any on-site swelling or temperature changes that might hinder the probe’s functionality (given that temperature fluctuations are prone to interfere with nanoscale devices [75,76,77,78]). Second, it protects haemophiliacs from the dangers that generally coincide with damage to epidermal tissues and other organ structures (assuming that zero-damage systems could be devised and proven effective). Third, the lack of immune response may also lead to the lack of a nervous system response to the probe’s entry and sustained presence. This latter point is crucial for enabling a “pure” setting whereby nervous system monitoring may be achieved, and therefore a more immediate understanding of whether there are differentiations within the nervous system for heat, pain, and other sensations specific to our sense of touch (including sensations picked up by the hair on our bodies).

Should this technique prove effective for ECM penetration without tissue damage resulting from long-term or multiple usage(s), the idea is to test whether BBB penetration would similarly be feasible utilising the same techniques. Given the dangers that would arise from a long-term tear or hole appearing on the BBB’s structure, such an experiment may ultimately prove to be ethically non-viable, however. As such, a more immediate short-term solution for cerebral monitoring and control would (theoretically) be viable through nervous system access via the spinal column as alluded to in Section 2 and portrayed in the referenced media [27,30]. Although the issue then arises that the temporal and spatial resolution for neuron activity would hardly be better than most of the techniques available to physicians today [32,33], making nano-driven neurological examination equally as impractical and therefore undesirable to pursue in greater depth.

Whether this system would ever be used to monitor the state of a child *in utero* is also something that will require careful consideration and may ultimately impact the early detection of pregnancy in women (to the benefit or detriment of any rights they may hold toward the abortion of the developing foetus). Given the potential benefits that would arise with *in utero* or *in vitro* biomonitoring of foeti when considering germline genetic manipulation or early-stage detection of developmental or other deadly defects, it is difficult to know without prior experimentation whether nanostructure penetration of developing tissue is safe. Arguably, if we can harmlessly enter the ECM and BBB, we should similarly be able to enter a placenta without causing harm to the mother or developing child. Given the extent to which ethical and moral arguments would consume that type of experimentation, it will be left for those other forums alongside whether the ability to detect or induce pregnancy impacts the rights held by women more generally.

The greatest challenge that researchers face once small-scale studies have been conducted on manufactured tissues is organism-based experimentation. Even if we assume that studies are conducted to test how nanostructures perform under accelerated heart rates or high blood pressure environments, there will still be concerns that nanoprobes and wires will accumulate throughout the bloodstream. Of course, it would be advisable that tests be conducted to display the potential impact of the presence of these structures in the smallest diameter veins present in the human form (coupled with the impact of these structures in plaque-coated veins). However, early developmental considerations will need to be given for how “loose” nanostructures can be destroyed before causing harm to appeal to critics against real-subject experimentation utilising these techniques. The struggle with those considerations is that different nanofabrication methods produce products that may or may not be optimal for ECM-breaching coupled with data transmission or nutrient transportation. Furthermore, not every structure can be equipped with a “kill” switch—which may entail that the areas where these structures are found undergo heating or magnetism that would be detrimental to the proper functioning of cells, tissues, and organs in the targeted area.

Of course, these considerations are part of the reason why commercial production and distribution of nanostructures are limited to lipid-based products or quantum dots—outside of those components that are used to power various larger electronic appliances. It is not for lack of manufacturing methods that nanostructures remain limited, but that their application in medicine is fraught with ethical and moral concerns that are not quickly resolved. Even if moves are made to develop a system whereby wirelessly imparted commands drive nanoprobes or nanorobots, the reality remains that our current technological abilities limit the ability for these devices to have functional computer systems that would be able to make independent decisions (including the ability to self-activate a “kill” command). This fact becomes incredibly complex when we consider that the internal structure of organisms possesses slight variances, as we can observe from noting the frame of another human (e.g., their height, build, sex). Even if nerves develop along a generally understood path, there is a greater-than-zero possibility that a given nerve cell will branch in an “incorrect” manner—which would cause it to develop through ligaments, for example. As a result, even attempting to develop a GPS requires that the internal structure of each new individual be completed before it would be helpful. Furthermore, there are complications that arise with maintaining an internal GPS of a patient’s body specific to the management of that data (from interoperability and accessibility more generally, to its long-term preservation and deletion).

Hence, there is a need to contemplate this technology’s “invasive” nature and how we may best mitigate potential harms to the patient with their implementation. Without thoroughly considering the benefits and detriments that this theoretical research poses, we cannot similarly consider how other advances in the anaesthesiologic world similarly impact patient populations and their preferences for accelerated recovery times via less invasive procedures.

## 4. Conclusions

In summary, several considerations will need to be made if researchers begin the development of medical nanostructure-centred artefacts as described. These go beyond those that are typically expected for other systems that monitor internal structures, given that the primary focus would be to interface with the CNS or PNS directly. Other considerations were described alongside those necessary to interface with nerve structures properly because internal structures cannot be considered independent from one another. Only approaching the subject from a single perspective neglects the reality that the CNS and PNS communicate with surrounding tissue structures, and that their homeostasis directly impacts the ability of other structures to maintain homeostasis. Such considerations may already be noted in the field of pain management but are essential to emphasise primarily because other disciplines may not take that understanding under consideration.

With that stated, it is hoped that the lack of a workable prototype is similarly understood by those reading this current piece. While nanoscale devices are gaining in sophistication and commercial ability as time progresses, much of the research being conducted for these devices are restricted to national laboratories and specialised research institutions that—by virtue of their status and focus—require a highly specialised set of skills, education, and experience, all of which this author lacks on account of their humanities-based research foci and access to said facilities. Nevertheless, it is hoped that this current work can inspire those with the means and talents to pursue the viability of the ideas espoused herein. Furthermore, it is hoped that a discourse on theoretical “compoundless anaesthesia” techniques of this nature can spur clinical practitioners into a similar mindset unbound by the current standards and norms of the field.

## Data Availability

Not applicable.
